# Spectral Characteristics of Square-Wave-Modulated Type II Long-Period Fiber Gratings Inscribed by a Femtosecond Laser

**DOI:** 10.3390/s21093278

**Published:** 2021-05-10

**Authors:** Xiaofan Zhao, Hongye Li, Binyu Rao, Meng Wang, Baiyi Wu, Zefeng Wang

**Affiliations:** 1 College of Advanced Interdisciplinary Studies, National University of Defense Technology, Changsha 410073, China; zhaoxiaofan_zxf@nudt.edu.cn (X.Z.); lihongye@nudt.edu.cn (H.L.); raobinyu@nudt.edu.cn (B.R.); wangmeng@nudt.edu.cn (M.W.); wubaiyi@nudt.edu.cn (B.W.); 2 Hunan Provincial Key Laboratory of High Energy Laser Technology, Changsha 410073, China; 3 State Key Laboratory of Pulsed Power Laser Technology, Changsha 410073, China

**Keywords:** long-period fiber gratings, higher-order harmonics, femtosecond laser

## Abstract

We study here the spectral characteristics of square-wave-modulated type II long-period fiber gratings (LPFGs) inscribed by a femtosecond laser. Both theoretical and experimental results indicate that higher-order harmonics refractive index (RI) modulation commonly exists together with the fundamental harmonic RI modulation in such LPFGs, and the duty cycle of a square wave has a great influence on the number and amplitudes of higher-order harmonics. A linear increase in the duty cycle in a series of square wave pulses will induce another LPFG with a minor difference in periods, which is useful for expanding the bandwidth of LPFGs. We also propose a method to reduce insertion loss by fabricating type II LPFGs without higher-order harmonic resonances. This work intensifies our comprehension of type II fiber gratings with which novel optical fiber sensors can be fabricated.

## 1. Introduction

LPFGs can realize the coupling between the copropagating core modes and cladding modes [1]. In the single-mode transmission region, the fundamental mode diverts to cladding modes after passing through an LPFG, and LPFGs can also play a role as mode converters in few-mode fibers. Since the first demonstration in the 1990s, LPFGs have been applied in many fields up to now, such as fiber sensors, fiber lasers, and so on [2]. Due to the high sensitivity of coupled cladding modes to the environment, LPFGs found their applications in RI sensing [3,4]. Furthermore, LPFGs were applied in temperature or stress detection [5,6,7,8]. Except for applications in fiber sensors, researchers applied LPFGs as spectral filtering devices in fiber lasers [9,10,11,12]. For example, LPFGs have been used to suppress the stimulated Raman scattering (SRS) effect in high-power fiber lasers, where core-guided SRS light can be stripped out of the core by inserting LPFGs, and the proportion of signal light in the output terminal can be increased [9,10,11]. By inscribing LPFGs in an erbium-doped fluoride glass fiber, Heck et al. proposed a new concept to mitigate the parasitic laser effect in mid-infrared fiber amplifiers [12].

Up to now, many methods have been utilized to fabricate LPFGs, including mechanical stress [13,14], electrical arc [15,16], CO_2_ laser [2,17], femtosecond laser [18,19], and so on. Exerting periodical mechanical stress on a few-mode fiber could induce coupling between two copropagating core-guided modes, but such LPFGs could persist when mechanical stress was released [20]. A periodic taped structure induced by an electrical arc or structuring a fiber with a CO_2_ laser could also realize the fabrication of LPFGs [21,22,23,24], but these LPFGs were relatively fragile because a few sections of the fiber became thinner just like microfibers [25,26,27]. UV lasers could inscribe relatively stable LPFGs by the direct-writing method or with the help of amplitude masks, but sufficient photosensitivity of fibers is necessary, so fibers should usually be hydrogen loaded in advance [28]. A femtosecond laser is another effective tool to fabricate LPFGs [29,30,31] with which the fiber is intact after fabrication, and the photosensitivity of fibers is not necessary. 

Recently, Heck et al. reported their investigations on type I LPFGs inscribed by a femtosecond laser, whose cross-RI modulation was positive (magnitude: 1 × 10^–4^) and took up the majority of the core area [32,33]. Type I LPFGs might degenerate in high-temperature environments. Another kind of LPFG inscribed by a femtosecond laser, namely type II LPFGs, is completely different. The RI profile is negative (magnitude: 1 × 10^–3^) and usually highly localized. Even when the temperature is more than 1000 °C, type II gratings can still keep their characteristics [34]. Their fine temperature tolerance makes LPFGs good candidates in high temperature sensing and high-power fiber laser systems. Square-wave-modulated type II LPFGs are easy to fabricate, but the insertion loss is relatively higher than that of type I, and the spectrum is in chaos. Up to now, few studies have interpreted their characteristics. 

In this paper, we investigate the characteristics of square-wave-modulated highly localized type II LPFGs inscribed by a femtosecond laser in detail. Both theoretical and experimental studies show that higher-order harmonic resonances coexist with fundamental frequency resonance in square-wave-modulated type II LPFGs, and the duty cycle of the square wave affects the amplitude and the number of higher-order harmonic resonances. With a linear increase in the duty cycle in a series of square wave pulses, resonances induced by a new LPFG of periods with little differences compared with the setting period occur. Based on the research results, a method to suppress higher-order harmonic resonances in type II LPFGs is proposed.

## 2. Theory

LPFGs could realize the coupling between two different modes (core-guided or cladding-guided modes) transmitted in the same direction. The resonance wavelength is decided by a phase-matching condition:

(1) $$\beta_{1}-\beta_{2}=m\frac{2\pi }{\Lambda }.$$

Here, *m* is the resonant order, *β*_1_ and *β*_2_ are the propagating constant of the two coupling modes, and Λ is the grating period of the LPFG. According to the relation of *β* = 2π*n_eff_*/*λ*, the resonant wavelength can be given by

(2) $$\lambda =(n_{eff,1}-n_{eff,2})\frac{\Lambda }{m}.$$

**Figure 1:**
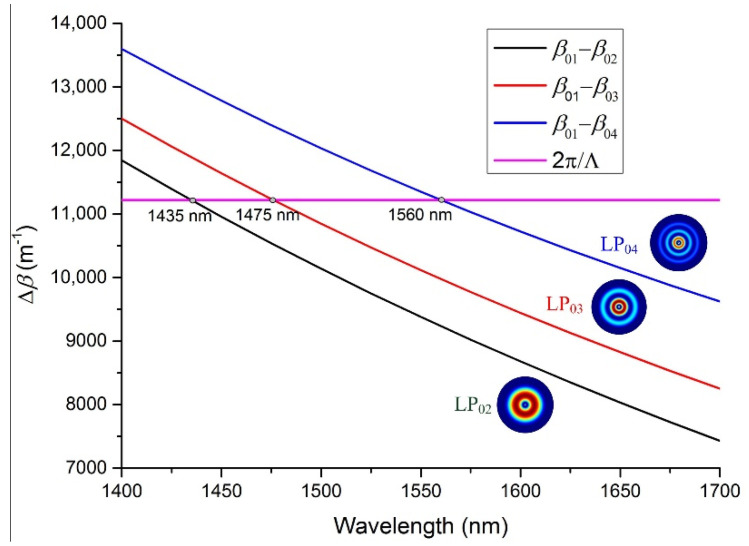
Phase-matching condition of the first-order harmonic resonance.

Here, *n_eff_*_,1_ and *n_eff_*_,2_ are the effective RI of the two different modes. For single-mode operation, the core-guided fundamental mode can only be coupled to cladding modes, and a bunch of resonant wavelengths appear in the transmission spectrum. Considering the symmetry, the fundamental mode mainly couples to axisymmetric cladding modes LP_0n_. Figure 1 shows the phase-matching condition of the first-order resonance (*m* = 1) when the grating period is 560 μm. The horizontal line represents 2π/Λ, and the intersection points define the resonant wavelengths. Simulation results indicate that LP_01_ mode couples to cladding mode LP_02_ at the wavelength of 1435 nm, to LP_03_ at 1475 nm, and LP_04_ at 1560 nm.

**Figure 2:**
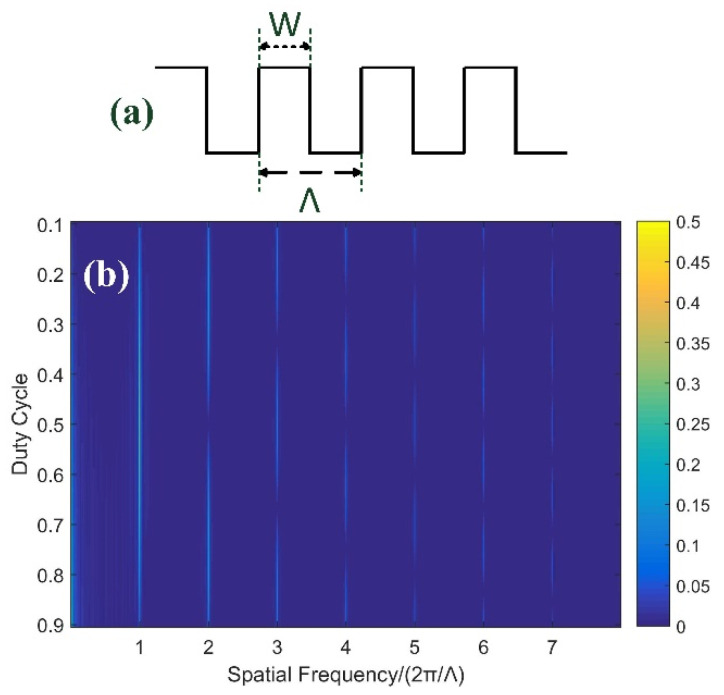
(**a**) Schematic of a square wave. (**b**) Spatial spectrum characteristics of a square wave with different duty cycles (color bar indicates the amplitude of each harmonic).

Except for the resonant points of LPFGs, the RI modulation function is another focus that has to be investigated in detail. For most femtosecond-laser-inscribed LPFGs, the RI modulation function along the fiber axis is in the form of a square wave within which harmonics cannot be ignored, especially for type II LPFGs. Each harmonic represents an LPFG with a different period. Moreover, the duty cycle of the square wave shows a great impact on the amplitude of harmonics. In practice, square wave modulation cannot be infinitely long (provided a finite number of periods). Figure 2a shows a typical square wave. Assuming n is the number of periods, Λ is the period, and W is the high period, the duty cycle is defined as the ratio of high period to the total period

(3) $$D=\frac{W}{\Lambda }.$$

The expression of finite length square wave can be written as

(4) $$Square(x)=A\times \sum_{n=1}^{N}rect\left(\frac{x-\frac{D\times \Lambda }{2}-(n-1)\times \Lambda }{D\times \Lambda }\right).$$

Here, *A* is the amplitude of square wave. To study its spatial spectrum characteristics, Fourier transform is carried out on this function:

(5) $$\begin{array}{l} F\left(Square(x)\right)=A\times \sum_{n=1}^{N}\int_{(n-1)\times \Lambda }^{(n-1)\times \Lambda +D\times \Lambda }e^{-jkx}dx\\ \begin{array}{cc} \; & \end{array}=A\times \frac{\left(1-e^{-jkD\Lambda })\times (1-e^{-jNk\Lambda }\right)}{jk\times (1-e^{-jk\Lambda })}. \end{array}$$

where *k* is the symbol of spatial frequency. Except *k* = 0, pole points of this function occur at *k* = 2π*m*/Λ (*m* = 1, 2, 3, 4, …), and the extremum of this function can also be found at these points. Figure 2b demonstrates the spatial spectrum of the square wave with different duty cycles when the number of the period is 72. It is obvious that not all harmonics coexist under any duty cycle, but it is not just fundamental harmonic that matters. For example, when the duty cycle is 50%, only odd-order harmonics can be excited, and the amplitude of even harmonics is zero. The amplitude of each harmonic varies with the duty cycle. At least we can say that in square-wave-modulated LPFGs, higher-order harmonic RI modulation can also impact the transmission spectrum.

**Figure 3:**
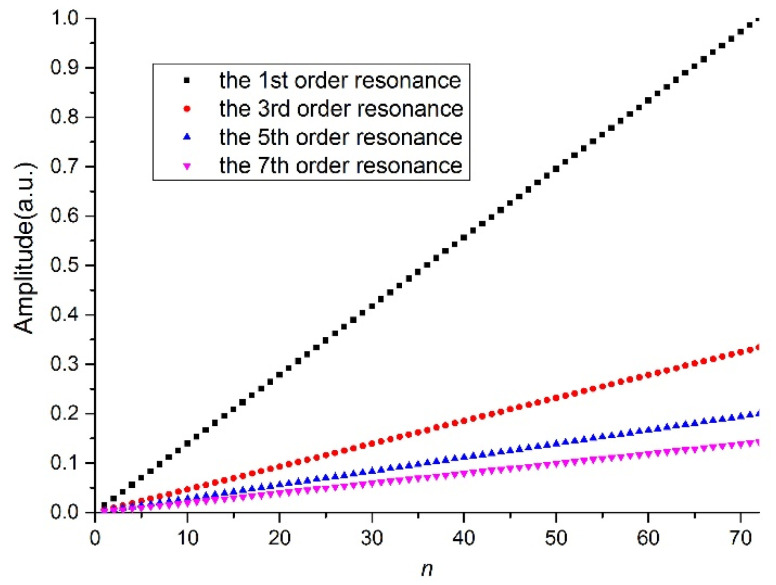
Amplitude of each resonance versus the number of periods.

Figure 3 shows the normalized amplitude of different harmonics versus the number of periods when the duty cycle is 50%. The amplitude of each harmonic increases linearly with the number of periods. The higher the order of the harmonic, the lower the amplitude. We can predict that in LPFG fabrication, the fundamental harmonic occurs even when the length of the LPFG is very short, but higher-order harmonics occur when the LPFG stretches to a certain length.

**Figure 4:**
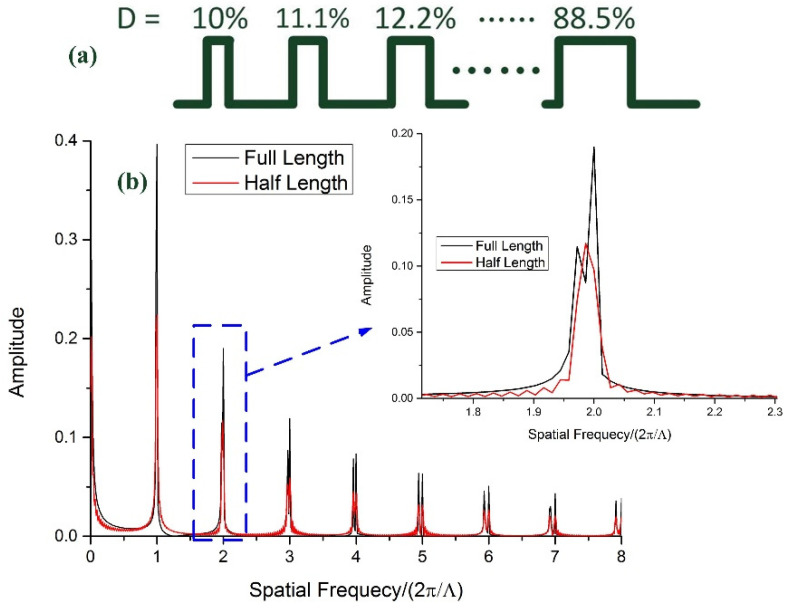
(**a**) Waveform of a square wave with a linearly growing duty cycle. (**b**) Spatial spectrum characteristics of a square wave with a linearly growing duty cycle.

Considering the situation where the duty cycle is no longer a constant in any part of a square wave sequence, the characteristics of the spatial spectrum show a large difference. For instance, the duty cycle grows linearly from 10% to 88.5% with the length of the square wave shown in Figure 4a (the total number of periods is 72):

(6) $$Square_{l}(x)=A\times \sum_{n=1}^{N}rect\left(\frac{x-\frac{D_{n}\times \Lambda }{2}-(n-1)\times \Lambda }{D_{n}\times \Lambda }\right).$$

(7) $$D_{n}=0.1+0.011(n-1).$$

Here, *D_n_* is the duty cycle of the *n*th square wave pulse (*n* = 1, 2, …, 72). The Fourier transform of Equation (4) can be written as:

(8) $$\begin{array}{c} F\left(Square_{l}(x)\right)=A\times \sum_{n=1}^{N}\int_{(n-1)\times \Lambda }^{(n-1)\times \Lambda +D_{n}\times \Lambda }e^{-jkx}dx\\ =A\times \frac{\left(1-e^{-j1.011k\Lambda })\times (1-e^{-jNk\Lambda })-e^{-j0.1k\Lambda }\times (1-e^{-jk\Lambda })\times (1-e^{-j1.011Nk\Lambda }\right)}{jk\times (1-e^{-jk\Lambda })\times (1-e^{-j1.011k\Lambda })}. \end{array}$$

In Equation (6), there are two series of pole points except *k* = 0. The first pole points series is represented as *k* = 2π*m*/Λ, and the second is *k* = 2π*m*/1.011Λ (*m* = 1, 2, 3, …). The spatial spectrum characteristics are demonstrated in Figure 4b. When the number of periods is 72 (full length), two series of harmonics occur in the spatial spectrum, and as the order of harmonics increases, the frequency difference also grows. Only two fundamental harmonic frequencies overlap with each other for the reason of small frequency differences. However, if the number of the period is half (36 periods), although two series of resonance frequencies are still obvious in higher-order harmonics, the spatial frequency difference is extremely small in the first- and second-order resonance region. In practical LPFG inscription, if the duty cycle increases with the length of the LPFG, one possible phenomenon we can observe is that another comb of dips grows up near the original second harmonic resonance wavelengths, and the bandwidth expands consequently.

## 3. Experiment

**Figure 5:**
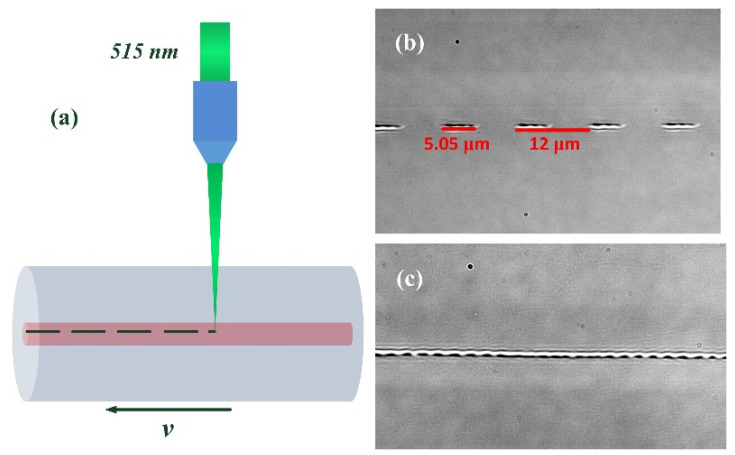
(**a**) Schematic of LPFG fabrication. (**b**) RI modulation generated point-by-point (magnification: 100×). (**c**) Microscope image of a square-wave-modulated LPFG (magnification: 100×).

A schematic inscription diagram for square-wave-modulated LPFGs is shown in Figure 5a. A femtosecond laser (Pharos Light Conversion) with 190 fs pulses, a central wavelength of 515 nm, and a repetition rate of 1 kHz s focused with an oil-free microscope objective (Mitutoyo) with a magnification of 100×. The pulse energy after the objective is about 150 nJ. An SMF (the core diameter is 8.2 μm, and the numerical aperture is 0.14) is fixed on a high-precision three-axis linear translation stage. During LPFGs fabrication, the horizontal speed *v* is set as 1000 μm/s, and the spacing between two successive points is 1 μm in theory. However, the cylindrical lens effect of fiber cannot be ignored in an oil-free inscription environment, the RI modulation generated by a pulse takes on an ellipsoid with its major axis aligning in the horizontal direction of fiber, as shown in Figure 5b. The length of the major axis is more than 3 μm when the pulse energy is 150 nJ; thus, the RI modulation generated by two successive pulses overlaps with each other, as shown in Figure 5c. Because the LPFG is highly localized, and if the refractive index modulation generated by a femtosecond laser is not high enough, the spectrum will not appear. We tried to place our LPFGs in a high-temperature (500 °C) furnace for more than 8 h. The spectra after cooling showed little difference compared to the spectra before high-temperature annealing. Thus, we confirm the LPFGs are type II.

**Figure 6:**
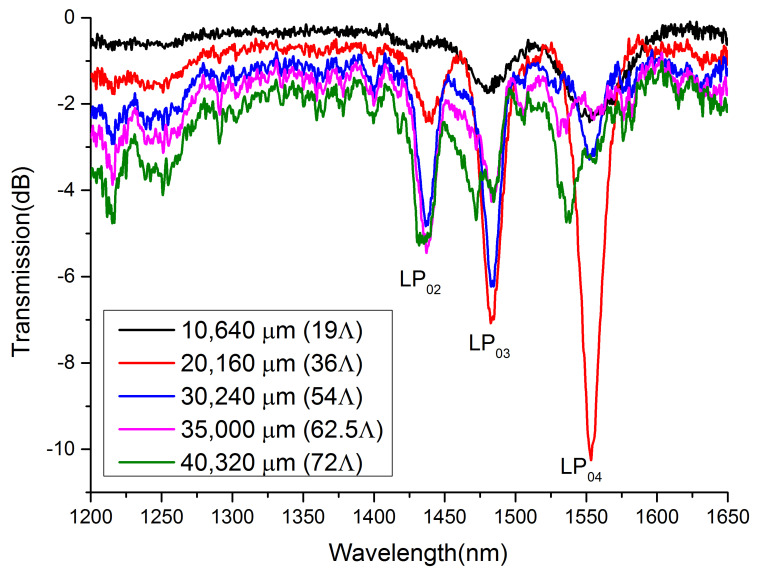
Spectrum evolution during LPFG fabrication (period: 560 μm; duty cycle: 50%. Insertion loss from 1200 to 1350 nm increases with the grating length).

The growth process of an LPFG is illustrated in Figure 6. The period is 560 μm, and the total length is 40,320 μm. The spectra of five different lengths are recorded in our experiment. As we discussed in Figure 3, only the LPFG stretches a certain length can higher-order harmonic resonance of the grating occur. In our experiment, when the length of the LPFG is longer than 20,160 μm, the higher-order harmonic resonance of the grating becomes discernible. With the increase in the LPFG length, the insertion loss goes up, as scattering loss induced by a femtosecond laser grows up in this process. The coupling between the fundamental mode and higher-order cladding mode (LP_04_) saturates prior to the lower-order cladding mode (such as LP_03_ and LP_02_). Because the LPFG does not strictly localize in the center of the core, the cross-RI profile takes on an axisymmetric rather than a circularly symmetric form; thus, birefringence becomes obvious when the LPFG is long. In the LPFG fabrication process, mode coupling is a dynamic process, for which energy in cladding modes may convert back to the fundamental mode when the grating reaches a certain length (this process is named overcoupling). As shown in Figure 6, after the overcoupling process, the resonance re-enhances, and birefringence occurs in the meantime.

**Figure 7:**
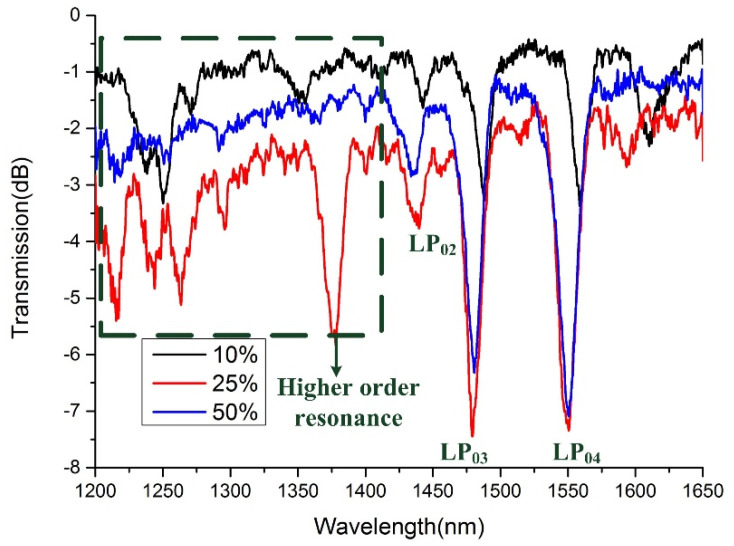
Transmission spectra of LPFGs with different duty cycles (insertion loss of the LPFG with a duty cycle of 10% is the smallest, and that of the LPFG with a duty cycle of 25% is the highest).

Figure 7 illustrates the transmission spectra of LPFGs with different duty cycles. The length of LPFGs is fixed as 40,320 μm, and the period of LPFGs is 560 μm. Thus, the number of periods is 72. Except for the first-order resonance, where the fundamental mode couples to the LP_02_, LP_03_, and LP_04_ cladding mode at around 1425, 1480, and 1550 nm, respectively, which is close to the theoretical analysis in Figure 1, and the error mainly comes from the neglect of material dispersion, the higher-order harmonic resonance of the grating is also visible. The occurrence of higher-order harmonic resonance creates chaos over the transmission spectrum of the LPFG. What has to be pointed out is that the higher-order resonance generated by the LPFG with a duty cycle of 50% is weaker than the other two LPFGs, which can be explained by what we discussed in Figure 2 that the harmonic number of square waves with a duty cycle of 50% is less than the other duty cycle.

**Figure 8:**
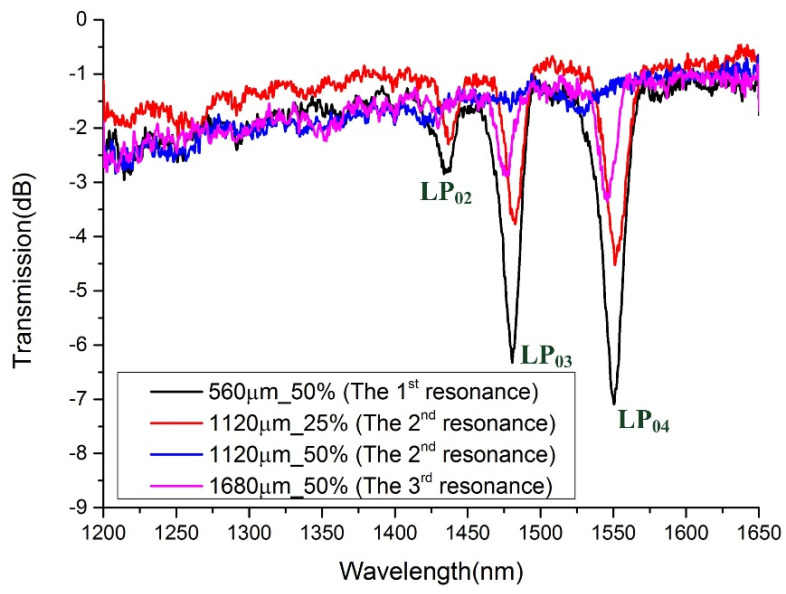
Transmission spectra of LPFGs with periods of 560, 1120, and 1680 μm (grating length: 40,320 μm. Resonant intensity decreases with the period, and when the duty cycle is 50%, the second-order harmonic resonance of the grating disappears).

Figure 8 demonstrates the transmission spectra of different-order resonances. The periods of the LPFGs are 560, 1120, and 1680 μm, which, respectively, correspond to the first-, second-, and third-order harmonic resonances generated by grating in the focusing wavelength band. The grating length is 40,320 μm regardless of the period. When the duty cycle is 50%, the second-order harmonic resonance of the grating disappears as the blue line in Figure 8 shows, but the second-order harmonic resonance of the grating is evident if the duty cycle is 25% (red line in Figure 8). The pink line performs the transmission spectrum when the period is 1680 μm and the duty cycle is 50%. The resonant intensity is relatively weak compared to the other situation, as the amplitude of the third-order harmonic resonance is only one-third of what it is in the first-order resonance.

**Figure 9:**
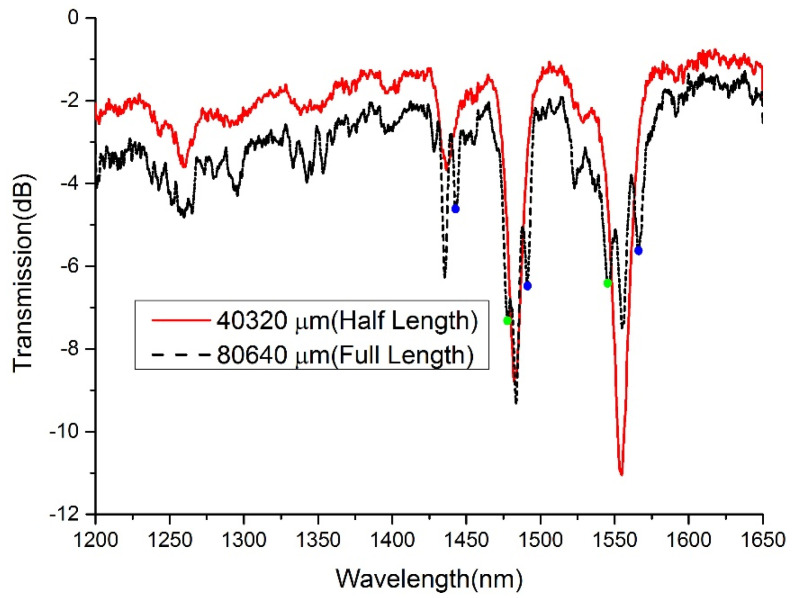
Spectrum evolution during LPFG fabrication (insertion loss increases with the grating length).

To testify the inference in Figure 4, an LPFG with different duty cycles (linearly increasing from 10% to 90%) in different periods is fabricated. Because the frequency difference is relatively small in the first-order resonance region, the period of the grating is set as 1120 μm to realize the second-order harmonic resonance of the grating in the focusing wavelength band, and the total length of the LPFG is 80,640 μm. Figure 9 illustrates the transmission spectrum of the LPFG during our fabricating process. When the grating length is 40,320 μm (half of the total length), the spectrum is similar to that depicted in Figure 6, where the second-order harmonic resonance of the grating takes up the majority, and the higher-order harmonic resonance of the grating shows up as well. However, the spectrum of the full-length LPFG is much more different. Except for the former resonant points, another series of dips (marked as blue points) appear. Meanwhile, the birefringence (marked as green points) also presents for the reason of the long grating length. These phenomena agree well with the aforementioned theoretical analysis. A derived LPFG that possesses different periods compared with the setting one is fabricated by linearly increasing the duty cycle of the time series pulse. The method shows the potential in bandwidth widening. 

The coupling characteristics of square-wave-modulated type II LPFGs are much more complex compared to those of type I, which provides more choices for the fiber sensing system. However, the existence of higher-order harmonic resonance of the grating disturbs the transmission spectrum and creates undesirable insertion loss, which forbids type II LPFGs as the fiber laser system application. Thus, a method to suppress the higher-order harmonic resonance of the grating in type II LPFGs is necessary. Considering that a square wave is composed of several harmonics and higher-order harmonics RI modulation contributes to higher-order harmonic resonances of the grating, if the RI modulation along with the fiber axis takes on a sinusoidal shape, the higher-order harmonic resonance of the grating cannot occur. To realize our assumption, the repetition frequency of the femtosecond laser should be high enough to make the RI modulation area generated by two successive pulses overlap with each other. The pulse energy is a time-varying sine wave, and the period of the sine wave equals the period of the LPFG. In this way, the RI modulation along the fiber axis takes on a sine-like shape (RI modulation is based on the nonlinear multiphoton absorption process, and the RI modulation along the fiber axis cannot be a strict sine-wave). The higher-order harmonic resonance of the grating may be largely suppressed by this method.

## 4. Conclusions

We investigated the coupling characteristics of square-wave-modulated type II LPFGs fabricated by a femtosecond laser. Both theoretical and experimental results show that except for the fundamental frequency resonance induced by a square wave, higher-order harmonic resonances are also significant in such LPFGs. The duty cycle shows a great effect on the amplitude and number of higher-order harmonic resonances of LPFGs. By varying the duty cycle in a series of square wave pulses, an LPFG with two different periods compared to the fabricated period can be derived, by which we can expand the bandwidth of the LPFG. The research work enhances our comprehension of type II fiber grating and shows potential in novelty sensor fabrication. A method to fabricate type II LPFGs with a higher-order harmonic resonance of the grating suppression is proposed. In the future, we will realize the fabrication of sine-wave-modulated type II LPFGs. In this way, the higher-order harmonic resonance of the grating can be avoided, and the insertion loss can be reduced. Type II grating is more suitable for applications of optical fiber sensors and fiber laser systems.

## Data Availability

Data sharing not applicable.
